# Cytolysis limits IFN-I and effector memory CD8 T cells after viral vector vaccination

**DOI:** 10.1016/j.omtn.2026.102885

**Published:** 2026-03-15

**Authors:** Pablo Penaloza-MacMaster

**Affiliations:** 1 Beth Israel Deaconess Medical Center, Center for Virology and Vaccine Research, 3 Blackfan Circle, E/CLS, Boston, MA 02215, USA

## Main text

Viral vectors are powerful tools in vaccine development, particularly because of their ability to induce robust CD8 T cell responses. However, how vector replicative capacity and cytolytic activity shape CD8 T cell responses remain incompletely understood. In this issue of Molecular Therapy Nucleic Acids, Ciancaglini et al. demonstrate that converting a cytolytic vesicular stomatitis virus (rVSV) vaccine vector into a non-cytolytic form (rVSVMq) enhances effector memory CD8 T cell responses and improves protection against infection.^1^ Mechanistically, this enhancement was linked to increased type I interferon (IFN-I) responses during CD8 T cell priming, establishing an interesting association between vector cytopathicity, IFN-I responses, and effector-memory CD8 T cell differentiation.

CD8 T cells are central to immunity against intracellular pathogens and tumors. To dissect how vector biology influences effector memory CD8 T cell responses, the authors compared two viral platforms: lymphocytic choriomeningitis virus-based vectors (rLCMV), which are non-cytolytic, and vesicular stomatitis virus-based vectors (rVSV), which are cytolytic due to the presence of the matrix protein that blocks the nuclear export of cellular RNAs to the cytosol resulting in cell death.^2^^,^^3^ Their data suggest that cytopathicity is not merely a safety consideration; rather, it fundamentally shapes IFN-I responses and subsequent CD8 T cell responses.

rLCMV vectors induced superior effector memory CD8 T cell responses than rVSV vectors, independent of glycoprotein pseudotyping. Using secreted nano-luciferase reporters, the authors demonstrate prolonged antigen expression in rLCMV-vaccinated mice compared to rVSV-vaccinated mice, suggesting that differences in cytolysis and antigen availability can affect the magnitude and differentiation of CD8 T cells. To directly test whether cytolysis was responsible, the authors utilized a non-cytolytic rVSV matrix quadruple mutant (rVSVMq). This modification enhanced effector memory CD8 T cells and conferred superior protection against *Listeria monocytogenes*, relative to the parental rVSV vector. Notably, these improvements were not attributable to increased antigen abundance, pointing instead to differences in IFN-I signaling.

A key insight of this study is that non-cytolytic replication amplifies systemic IFN-I responses. rVSVMq induced substantially higher IFN-I responses than cytolytic rVSV. Transcriptional profiling of CD8 T cells revealed enrichment of IFN-stimulated gene signatures in rVSVMq-vaccinated mice, accompanied by sustained effector differentiation. Blocking IFN-I/IFNAR signaling reduced effector memory CD8 T cell responses following vaccination with rVSVMq or rLCMV, suggesting a role for IFN-I. Adoptive transfer experiments further demonstrated that IFN-I signaling acts directly on CD8 T cells to promote their expansion and differentiation. These data reinforce the concept of IFN-I as a critical “signal 3” that cooperates with TCR and costimulatory signals during priming and suggest that non-cytolytic vectors facilitate spatiotemporal integration of TCR and IFN-I signals following viral vector vaccination.

Historically, IFN-I has been shown to be essential for antiviral defenses, but various reports, including some by the Pinschewer laboratory, have shown that it can also be detrimental during antiviral responses.^4^^,^^5^^,^^6^^,^^7^^,^^8^^,^^9^ This study adds important knowledge to the field by improving the understanding of how viral vector cytopathicity, antigen availability, and IFN-I dictate CD8 T cell responses following viral vaccination. By eliminating matrix protein-mediated host shutoff, rVSVMq preserves infected cell integrity long enough to permit sustained antigen presentation and IFN-I production that potentiates effector memory CD8 T cell responses.

Interestingly, enhancement of CD8 T cell responses with the non-lytic VSV vector was not associated with improved antibody responses. This divergence highlights that optimizing viral vectors for cellular immunity may rely on different rules than optimizing for humoral immunity. Such findings have implications for vaccinology. First, they identify cytopathicity as a modifiable parameter that influences not only safety but also CD8 T cell responses. Second, they position IFN-I as a locally integrated differentiation signal whose spatial and temporal dynamics critically determine memory CD8 T cell fate. The side-by-side comparison of cytolytic and non-cytolytic VSV platforms may also inform improvements to existing rVSV-based vaccines, including those used against Ebola virus.

Future studies should determine whether similar principles apply to other viral vector platforms, such as adenoviral, poxviral, or the recently characterized Orf virus-based^10^ vectors. Moreover, the translational relevance of enhancing IFN-I signaling must be carefully evaluated in humans, where excessive IFN-I responses may contribute to reactogenicity following vaccination. Looking ahead, rational viral vector vaccine design may increasingly rely on deliberate tuning of the vector’s life cycle for maximizing specific arms of the adaptive immune response.

## Declaration of interests

P.P.-M. is a consultant for Thyreos Vaccines. P.P.-M.’s laboratory is funded in part by the NIH and AAI.
